# Supplementary material for: GABA-Positive Astrogliosis in Sleep-Promoting Areas Associated with Sleep Disturbance in 5XFAD Mice
Source: Int J Mol Sci. 2023 Jun 2;24(11):9695. doi: 10.3390/ijms24119695 (PMC10253883; doi:10.3390/ijms24119695)
Supplement: Supplementary file 1 [file ijms-24-09695-s001.zip › ijms-2391474-supplementary.pdf]

Supplementary materials

Victor James Drew, Mincheol Park, Tae Kim

Table S1. Immunohistochemistry antibody list

Antibody	Species	Catalog #	Company	Dilution
Primary				
Anti-nNOS antibody	Mouse	Sc-5302	Santa Cruz Biotechnology, Dallas, TX	1:500
Anti-Choline acetyltransferase antibody	Goat	AB144P	Merck, Darmstadt, Germany	1:500
Anti-Galanin antibody	Goat	NBP1-45217	Novus Biologicals, Centennial, CO	1:500
Anti-GABA antibody	Guinea pig	AB175	Merck, Darmstadt, Germany	1:200
Anti-GFAP antibody	Rabbit	Z033429	Aligent, Santa Clara, CA	1:500
Anti-GABRD antibody	Rabbit	PA5-101598	Thermo Fisher Scientific, Waltham, MA	1:500
Secondary				
AlexaFlour-488 (anti-goat) IgG antibody	Donkey	A-11055	Thermo Fisher Scientific, Waltham, MA	1:500
AlexaFlour-488 (anti-mouse) IgG antibody	Donkey	A21202	Thermo Fisher Scientific, Waltham, MA	1:500
AlexaFlour-555 (anti-rabbit) IgG antibody	Donkey	A31572	Thermo Fisher Scientific, Waltham, MA	1:500
AlexaFlour-647 (anti-guinea pig) IgG antibody	Donkey	703-545-155	Jackson ImmunoResearch Laboratories, Inc., West Grove, PA	1:500

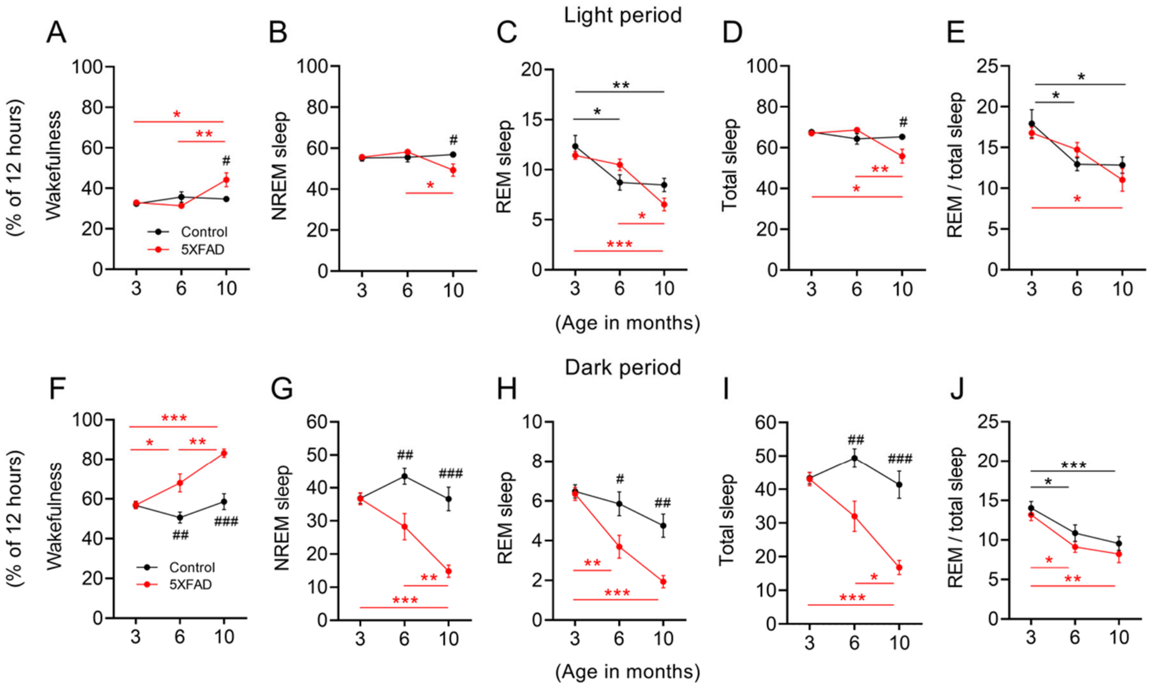

Figure S1. 5XFAD mice exhibit diminished NREM and REM sleep duration with age. (**A–E**) Total recording time during light period spent in wakefulness, NREM, REM, total sleep and REM/total sleep for control and 5XFAD mice of 3, 6 and 10 months of age. (**F–J**) Total recording time during dark period spent in wakefulness, NREM, REM, total sleep and REM/total sleep for control and 5XFAD mice of 3, 6 and 10 months of age. Data were presented as mean ± SEM (n = 10 - 12 mice per group). $^{*}p < 0.05$, $^{**}p < 0.01$, and $^{***}p < 0.001$ in red (5XFAD) and black (control) by ANOVA or Kruskal-Wallis among age groups. $^{\#}p < 0.05$, $^{\#\#}p < 0.01$, $^{\#\#\#}p < 0.001$ by independent t-test or Mann-Whitney u test between genotypes. *Abbreviations*: ANOVA, analysis of variance; EEG, electroencephalogram; NREM, non-rapid eye movement; REM, rapid eye movement

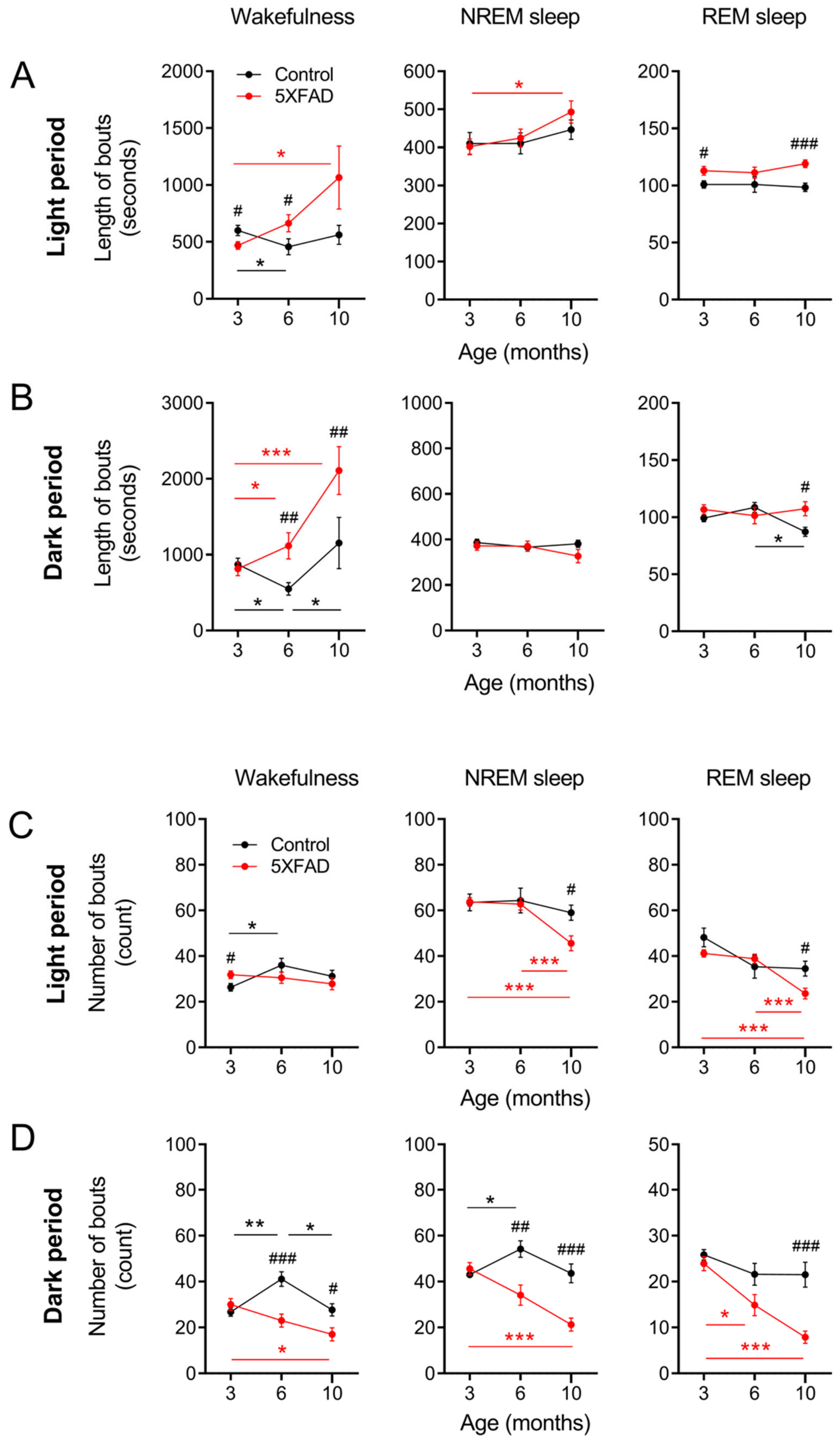
Wakefulness
NREM sleep
REM sleep
A
Light period
Length of bouts (seconds)
Control
5XFAD
Age (months)
B
Dark period
Length of bouts (seconds)
Age (months)
Wakefulness
NREM sleep
REM sleep
C
Light period
Number of bouts (count)
Control
5XFAD
Age (months)
D
Dark period
Number of bouts (count)
Age (months)

Figure S2. 5XFAD mice exhibit altered sleep bout characteristics with age. (**A**) Bout lengths during light period for wakefulness, NREM, and REM sleep for control and 5XFAD mice of 3, 6 and 10 months of age. (**B**) Bout lengths during dark period for wakefulness, NREM, and REM sleep for control and 5XFAD mice of 3, 6 and 10 months of age. (**C**) Bout counts during light period for wakefulness, NREM, and REM sleep for control and 5XFAD mice of 3, 6 and 10 months of age. (**D**) Bout counts during dark period for wakefulness, NREM, and REM sleep for control and 5XFAD mice of 3, 6 and 10 months of age. Data were presented as mean ± SEM (n = 10 - 12 mice per group). $^{*}p < 0.05$, $^{**}p < 0.01$, and $^{***}p < 0.001$ in red (5XFAD) and black (control) by ANOVA or Kruskal-Wallis among age groups. $^{\#}p < 0.05$, $^{\#\#}p < 0.01$, $^{\#\#\#}p < 0.001$ by independent t-test or Mann-Whitney u test between genotypes. *Abbreviations*: ANOVA, analysis of variance; EEG, electroencephalogram; NREM, non-rapid eye movement; REM, rapid eye movement

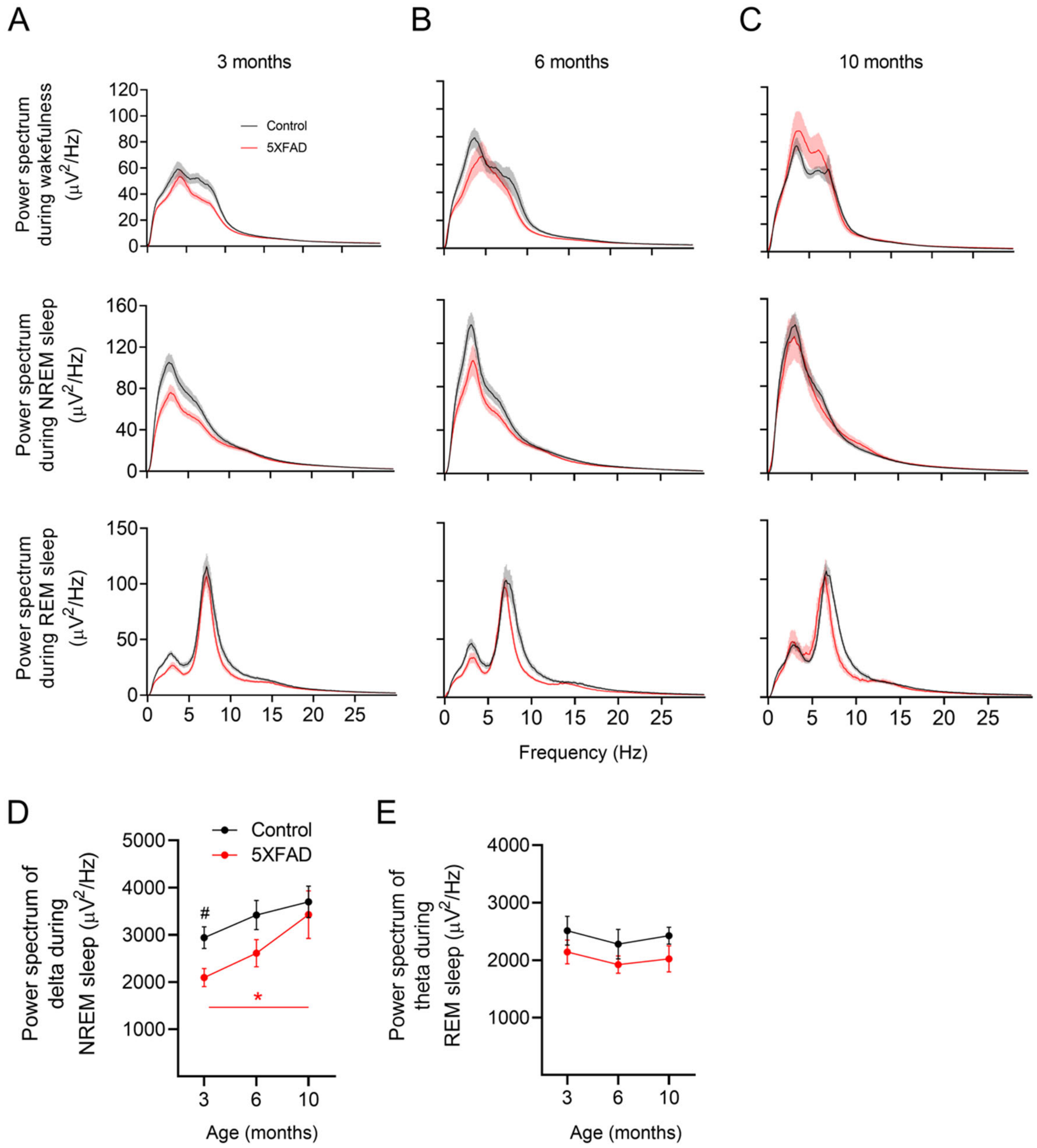

Figure S3. 5XFAD mice exhibit increased NREM delta power with age. (**A–C**) Full power spectra of 24-hour EEG recording session for wakefulness, NREM and REM sleep for control and 5XFAD mice of 3, 6 and 10 months of age, respectively. (**D**) Delta power during NREM sleep of control and 5XFAD mice at all age groups. (**E**) Theta power during REM sleep of control and 5XFAD mice at all age groups. Data were presented as mean ± SEM (n = 10 - 12 mice per group). *$p < 0.05$, in red (5XFAD) and black

(control) by ANOVA or Kruskal-Wallis among age groups (panels D and E). $^{\#}p < 0.05$, by independent t-test or Mann-Whitney u test between genotypes (panels D and E). *Abbreviations*: ANOVA, analysis of variance; EEG, electroencephalogram; NREM, non-rapid eye movement; REM, rapid eye movement

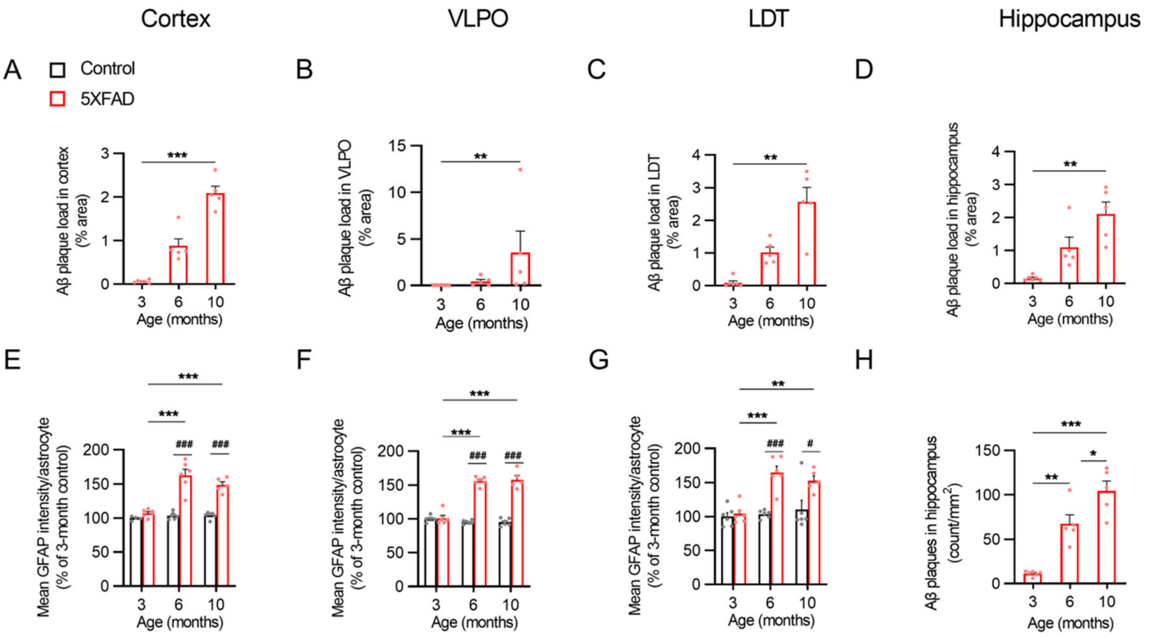

Figure S4. Regional increases in Aβ and astrocytosis in 5XFAD mice. (**A**) Aβ plaque load in the cortex of 5XFAD mice. (**B**) Aβ plaque load in the VLPO of 5XFAD mice. (**C**) Aβ plaque load in the LDT of 5XFAD mice. (**D**) Aβ plaque load in the hippocampus of 5XFAD mice. (**E**) The GFAP intensity observed in the cortex. (**F**) The GFAP intensity observed in the VLPO. (**G**) The GFAP intensity observed in the LDT. (**H**) The number of Aβ plaques per area unit of the hippocampus. Black and red bars indicated the mean value and raw data in control and 5XFAD mice, respectively. Each dot represents the raw data. Data were presented as mean ± SEM (n = 5-6 mice per group). $^{*}p < 0.05$, $^{**}p < 0.01$, $^{***}p < 0.001$ between ages, $^{\#}p < 0.05$, $^{\#\#\#}p < 0.001$ between genotypes by one-way ANOVA with Kruskal-Wallis post hoc test (panels A–D), and two-way ANOVA test with Tukey post hoc test (panels E–G),.

Abbreviations: ANOVA, analysis of variance; EEG, electroencephalogram; GABA, gamma aminobutyric acid; GFAP, glial fibrillary acidic protein; HIP, hippocampus; LDT, laterodorsal tegmentum; nNOS, neuronal nitric oxide synthase; NREM, non-rapid eye movement; REM, rapid eye movement; VLPO, ventrolateral preoptic area

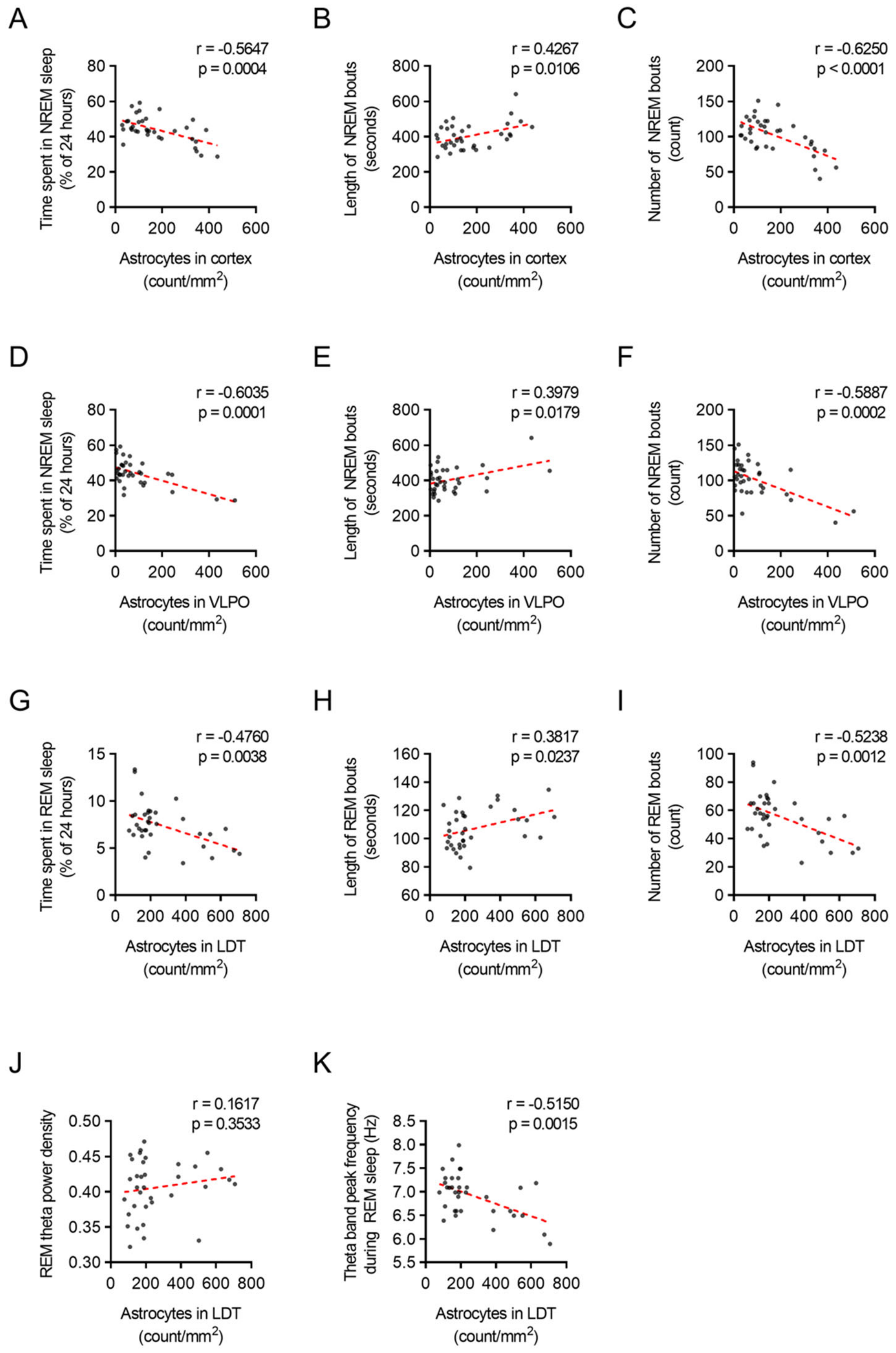

Figure S5. Sleep disturbance correlates with elevated astrocyte counts. (**A**) The correlation between the ratio of number of astrocytes in the cortex and NREM recording time (%). (**B**) The correlation between the ratio of number of astrocytes in the cortex and NREM bout length (%). (**C**) The correlation between

the ratio of number of astrocytes in the cortex and NREM bout count (%). (**D**) The correlation between the ratio of number of astrocytes in the VLPO and NREM recording time (%). (**E**) The correlation between the ratio of number of astrocytes in the VLPO and NREM bout length (%). (**F**) The correlation between the ratio of number of astrocytes in the VLPO and NREM bout count (%). (**G**) The correlation between the ratio of number of astrocytes in the LDT and REM recording time (%). (**H**) The correlation between the ratio of number of astrocytes in the LDT and REM bout length (%). (**I**) The correlation between the ratio of number of astrocytes in the LDT and REM bout count (%). (**J**) The correlation between the ratio of number of astrocytes in the LDT and REM theta peak frequency (Hz). Black and red bars indicated the mean value and raw data in control and 5XFAD mice, respectively. Each dot represents the raw data. n = 5-6 mice per group). Each dot represents the raw data. Data were presented as mean ± SEM (n = 5-6 mice per group) by Pearson correlation test (panels **A**-**K**). *Abbreviations*: ANOVA, analysis of variance; EEG, electroencephalogram; GABA, gamma aminobutyric acid; GFAP, glial fibrillary acidic protein; HIP, hippocampus; LDT, laterodorsal tegmentum; nNOS, neuronal nitric oxide synthase; NREM, non-rapid eye movement; REM, rapid eye movement; VLPO, ventrolateral preoptic area

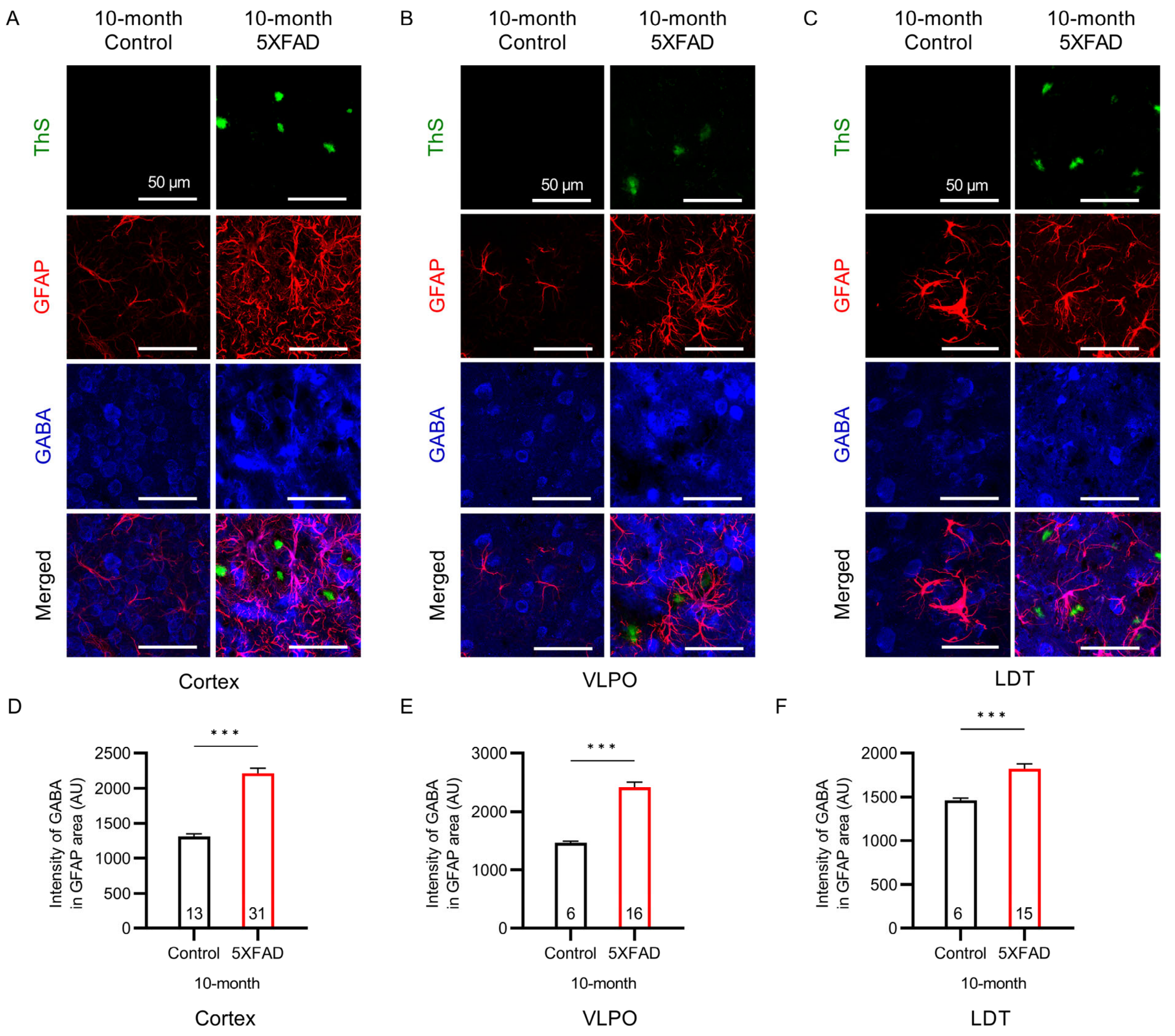

Figure S6. Enhanced magnification histology images and GABA intensity in GFAP area. Representative images of GABA-positive reactive astrocytes near the Aβ plaques in the cortex (**A**), VLPO (**B**) and LDT (**C**) of 10-month-old control and 5XFAD mice. Scale bar indicates 50 μm. Mean intensity of GABA in GFAP-positive areas in the cortex (**D**), VLPO (**E**) and LDT (**F**) based on representative images. Control and 5XFAD mice represented by black and red bars, respectively. Numbers at the base of each bar represent the number of astrocytes in representative images corresponding to respective brain region. Data were presented as mean ± SEM. Representative images were obtained using a single control and 5XFAD mouse. ***p <

0.001 by independent t-test (panels D–F). Abbreviations: GABA, gamma aminobutyric acid; GFAP, glial fibrillary acidic protein; LDT, laterodorsal tegmentum; VLPO, ventrolateral preoptic area.
